# Digital twin of the ocean as a catalyst for blue economy innovation

**DOI:** 10.1093/nsr/nwag012

**Published:** 2026-01-20

**Authors:** Fei Chai, Qiang Deng, Minhan Dai, Xiaoyi Wang, Joanna Staneva, Swadhin K Behera, Marina Tonani, Jian Liu, Zhaoyuan Yu, Zhong Peng

**Affiliations:** State Key Laboratory of Marine Environmental Science, College of Ocean and Earth Sciences, Xiamen University, China; Fujian Ocean Innovation Center, China; State Key Laboratory of Marine Environmental Science, College of Ocean and Earth Sciences, Xiamen University, China; Fujian Ocean Innovation Center, China; State Key Laboratory of Marine Environmental Science, College of Ocean and Earth Sciences, Xiamen University, China; Fujian Ocean Innovation Center, China; State Key Laboratory of Marine Environmental Science, College of Ocean and Earth Sciences, Xiamen University, China; Institute of Coastal Systems-Analysis and Modeling, Helmholtz-Zentrum Hereon, Germany; Application Laboratory (APL), Research Institute for Value-Added-Information Generation (VAiG), Japan Agency for Marine-Earth Science and Technology (JAMSTEC), Japan; Mercator Ocean International, France; Southern Marine Science and Engineering Guangdong Laboratory (Zhuhai), China; School of Geography and Ocean Science, Ministry of Education Key Laboratory for Coast and Island Development, Nanjing University, China; State Key Laboratory of Climate System Prediction and Risk Management (Nanjing Normal University), China and Jiangsu Center for Collaborative Innovation in Geographical Information Resource Development and Application, China; State Key Laboratory of Estuarine and Coastal Research, Institute of Eco-Chongming, East China Normal University, China

The global ocean economy is valued at ∼$2.5 trillion per year, larger than the size of the world’s seventh-largest economy. The traditional focus of the ocean economy has been economic maximization that frequently harms marine ecosystems and jeopardizes the livelihoods of dependent communities, while the blue economy promotes a sustainable marine-based economic model that harmonizes growth, ecological preservation, and social equity [1]. The blue economy is experiencing rapid growth and playing an increasingly vital role in the global socio-economic landscape. However, as maritime activities become increasingly complex, traditional governance approaches face limitations in real-time monitoring, cross-domain collaboration, and decision-making efficiency, necessitating the adoption of advanced digital technologies.

The Digital Twin of the Ocean (DTO) represents a transformative advancement in ocean science, leveraging powerful digital twin technology to create dynamic virtual replicas of marine systems [2]. By continuously integrating real-time observational data and sophisticated modelling, the DTO facilitates integrated and data-driven decision-making to promote the sustainable use of marine resources and protection. By providing a robust analytical framework to address critical operational questions, including immediate (What now?), prospective (What next?), and hypothetical (What if?) scenarios, it ultimately enhances organizational decision-making capabilities [3]. This capability is crucial for balancing socio-economic development with ecological conservation, thereby advancing the ‘blue transformation’ advocated by the UN Decade of Ocean Science for Sustainable Development (2021–2030) [4] and Sustainable Development Goal 14 [5].

The UN Ocean Decade highlights the importance of sharing and managing ocean data, information, and knowledge, and has launched the program DITTO to establish best practices, standards, and a framework for DTO [6]. The EU also has launched a program (EU DTO) to advance the rapid evolution of DTO technology and make ocean information readily accessible to researchers, entrepreneurs, local authorities, and citizens alike [7]. It is implemented through a collaborative network of specialized projects, each dedicated to distinct domains such as ecological monitoring, public data infrastructure, advanced ocean modeling, and socio-ecological research.

## ESSENTIAL ARCHITECTURE OF DTO

**Figure 1.**
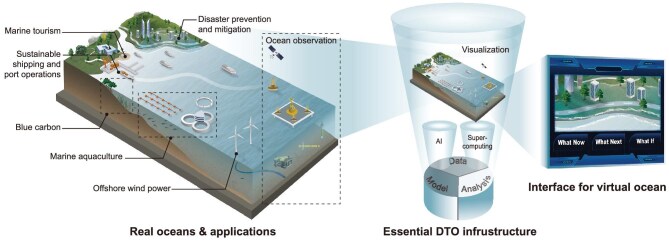
Conceptual framework of the digital twin system for the blue economy. (a) The DTO system enables diverse applications in the blue economy, including marine aquaculture, offshore wind power, sustainable shipping and port operations, disaster prevention and mitigation, blue carbon and marine tourism. (b) The core architecture of the DTO framework is built on Data, Model, Analysis, and Visualization modules, powered by AI and supercomputing technologies. (c) Through an AI-driven intelligent human-machine interface, the DTO system could provide real-time insights, predictive analytics, and scenario simulations, addressing key user queries: ‘What is happening now?’, ‘What will happen next?’, and ‘What if?’ scenarios.

The architecture of a DTO system typically adopts a hierarchical, multi-module framework [2]. Each functionally specialized module maintains operational independence while enabling synergistic interoperability across modules, thereby supporting comprehensive end-to-end applications in marine environments, underpinned by advanced AI and supercomputing capabilities (Fig. 1). While most current AI models in ocean science focus on environmental prediction, the development of specialized large language models (LLMs) expert in diverse ocean tasks, from scientific research to resource management, is a critical gap that must be filled in order to construct a comprehensive DTO.

In the ‘Data’ module, ‘Acquisition’ aggregates and stores multi-source marine data from satellite remote sensing, *in-situ* sensor networks, research vessels, autonomous buoys, and numerical simulations; ‘Processing’ implements data cleaning and format standardization; ‘Fusion and Embedding’ ensure data consistency and interoperability across heterogeneous sources with spatiotemporal alignment. The performance of data fusion hinges on the coordinated design of ocean observing systems [8], underpinned by interoperability standards. These standards provide unified specifications for data formats, interfaces, semantics, and architecture to integrate disparate marine data sources, models, and applications. As the core connective tissue of the DTO, data fusion must balance heterogeneity, physical consistency, and real-time performance, fostering an integration of ocean dynamics, statistics, and AI. Consequently, the field is transitioning from traditional numerical assimilation to an end-to-end, hybrid framework supported by edge computing and global collaboration.

In the ‘Model’ module, ‘Simulation’ enables dynamic, high-fidelity modeling of marine environments by integrating multi-source data; ‘Prediction’ combines data-driven and physics-informed forecast models, dynamically adjusted with the feedback from observational data, to accurately anticipate marine phenomena; ‘Assessment’ provides quantitative socio-economic value evaluation for environmental events, human activities, and management policies, to improve governance strategies.

In the ‘Analysis’ module, ‘Description’ provides statistical characteristics and spatial-temporal distribution features of various datasets; ‘Diagnosis’ examines why certain patterns or anomalies occur by identifying correlations, causations, and underlying factors; ‘Decision Optimization’ applies advanced mathematical modeling and AI to identify the best possible actions under given constraints, enhancing efficiency and outcomes in marine operations.

The ‘Visualization & Interactive’ module employs interactive visualization tools to intuitively present ocean states and forecast products, while its AI-powered natural language processing (NLP) interface enhances human–machine interaction, significantly reducing the technical barriers for non-specialists.

Traditional marine information platforms typically function like digital photo albums, offering static archives of historical and current ocean data for users to browse and retrieve. In contrast, the DTO operates as a high-fidelity, interactive flight simulator. It not only visualizes real-time conditions but also forecasts future scenarios and tests systemic responses to events such as storms or policy changes. Fundamentally, the DTO represents a paradigm shift from a passive data repository to an active, predictive decision-support engine. Enhanced by advanced AI, it can autonomously coordinate observation equipment, execute simulations, and analyze results, bringing the ocean system to life within a dynamic virtual environment.

## DTO APPLICATIONS FOR THE BLUE ECONOMY

DTO provides full life-cycle management support for blue economy sectors, covering planning, construction, operation, maintenance, and decommissioning. This holistic integration ensures that marine industrial activities are optimized for efficiency, resilience, and sustainability. Although different applications may prioritize different components, all architectural elements of the DTO are essential.

***Marine aquaculture***. DTO enables real-time tracking of key water quality parameters, including temperature, salinity, and oxygen, along with weather and current conditions, providing early warnings to farmers about harmful events such as algal blooms or hypoxia. Integrated IoT sensors continuously monitor fish behavior, feeding activity, and signs of disease, supporting optimized feeding strategies and stocking densities [9]. Digital models are used to monitor structural wear on cages and conduct virtual stress tests, evaluating infrastructure resilience under extreme conditions like storms or strong currents. Additionally, a sustainability compliance module ensures adherence to environmental regulations by simulating waste dispersion and assessing ecosystem impacts, thereby guiding responsible site selection to minimize the ecological footprint.

***Offshore wind power***. By simulating decades of ocean and weather conditions, such as waves, currents, winds, water depth, and seabed sediment properties, DTO supports precise site selection and comprehensive feasibility analysis [10]. Structural health is continuously monitored using digital replicas of monopiles or jackets, which track corrosion rates and predict fatigue life under combined wind and wave loads. The system also enables predictive maintenance by identifying bearing wear patterns weeks in advance of potential failure and optimizing vessel dispatch for repairs during favorable weather windows. Furthermore, it models dynamic stresses on cables caused by tidal currents and fishing activities, predicting possible fault locations with high accuracy.

***Sustainable shipping and port operations***. DTO integrates live AIS data, real-time weather forecasts, hydrodynamic models, and wave models to dynamically simulate and optimize voyage routes in response to changing environmental and regulatory conditions. This enables significant reductions in fuel consumption while avoiding hazardous sea states [11]. Similarly, in port management, DTO frameworks synthesize real-time data on vessel traffic, berth availability, cargo operations, and meteorological and oceanographic conditions to optimize berthing schedules. Operators can also assess the potential impacts of infrastructure modifications, such as new quay configurations or dredging strategies, before implementing physical changes [12].

***Disaster prevention and mitigation***. DTO is revolutionizing marine disaster management by shifting traditional reactive approaches to proactive and predictive strategies [13]. It employs high-resolution simulations of wind-wave-current interactions to forecast the impacts of typhoons and storm surges, delivering critical early warnings for risk of coastal inundation. By integrating satellite data, *in-situ* sensors, and ecosystem models, DTO can monitor the development of harmful algal blooms and predict the dispersion paths of toxins, thereby supporting timely fishery closures and water intake management. In the event of oil spills, the technology simulates spill trajectories under diverse oceanic and atmospheric conditions to optimize containment planning and evaluate cleanup strategies prior to deployment. Furthermore, DTO contributes to long-term coastal resilience planning by predicting sediment transport patterns under climate change scenarios and virtually assessing the effectiveness of mitigation structures, such as breakwaters and artificial reefs.

***Blue carbon***. DTO is transforming complex natural processes into quantifiable and verifiable climate assets. It enables comprehensive carbon stock assessments by integrating satellite imagery, drone surveys, and sediment core data, producing detailed 3D carbon storage maps while substantially reducing estimation uncertainties. DTO can simulate tidal hydrology and sediment dynamics to identify optimal sites for mangrove planting and forecast carbon sequestration potential. Furthermore, it strengthens carbon credit verification by providing tamper-proof blockchain-based documentation of carbon flux data and automating the MRV (measurement, reporting, and verification) processes for blue carbon offset projects. With these capabilities, DTO systems are positioned to become indispensable tools for verifying nature-based climate solutions and directing billions in climate finance to coastal communities worldwide [14].

***Marine tourism***. DTO creates immersive, data-driven digital replicas of coastal destinations and marine ecosystems, enhancing visitor engagement while supporting the sustainable management of fragile environments [15]. Through augmented reality interfaces, it enriches on-site experiences by overlaying information about historical shipwrecks and marine biodiversity onto mobile devices, enabling real-time species identification during snorkeling and diving activities. Additionally, DTO supports the preservation of coastal heritage by building detailed digital archives of eroding archaeological sites and providing virtual access to otherwise inaccessible submerged ruins. It also assists in marine park capacity planning by simulating visitor flow impacts on sensitive ecosystems and evaluating carrying capacity scenarios for new tourism infrastructure.

## CHALLENGES AND FUTURE DIRECTIONS OF DTO

The realization of a DTO relies on the convergence of multiple enabling technologies, each of which presents its own set of challenges. While substantial progress has been made in ocean observation systems, modeling, and AI integration, several technological gaps must still be addressed before DTO can fully support a sustainable blue economy.

Advanced data processing capabilities are necessary to integrate and manage diverse, multi-source, heterogeneous oceanographic datasets, which include not only massive surface observations but also sparse interior measurements. Integrated ocean-atmosphere-wave-biogeochemical models face challenges in accurately resolving nearshore and estuarine processes, as well as fine-scale physical–biological interactions. These challenges are compounded by high computational demands, raising concerns around high-performance and intelligent computing with energy efficiency. Furthermore, standardized frameworks and robust cybersecurity protocols are essential for data governance, to protect intellectual property, ensure privacy, and promote equitable access. Nations may withhold data for sovereignty, while regions lacking resources cannot contribute or benefit equally. This creates data silos and asymmetrical capabilities, preventing a truly global, collaborative system. However, integrating blockchain with Federated Learning presents a viable path forward. By combining Federated Learning’s privacy-preserving model training with blockchain’s transparent incentive mechanisms, these technologies enable secure and equitable global collaboration without the need to share raw data.

To address these challenges, the development of DTO must prioritize several key directions. To realize the full potential of DTO, a unified mathematical framework is essential. It must handle the high-dimensional, heterogeneous nature of ocean data by leveraging techniques like hypercomplex signal processing to improve data fusion and AI interpretability. Grounded in theories from Bayesian inference to constraint hypergraphs, this framework is pivotal for transcending current fragmentation, standardizing data integration, and enabling cross-disciplinary, scalable ocean intelligence. Hybrid modeling, which integrates data-driven machine learning with physics-based numerical approaches, can enhance predictive accuracy while preserving physical consistency and adaptability. Computational efficiency may be strengthened through edge-cloud computing architectures that balance processing loads and improve responsiveness. Interdisciplinary collaboration across oceanography, computer science, and AI is essential to foster innovation, while standardized protocols and open sharing mechanisms will support global interoperability and reproducibility.

Realizing DTO’s transformative potential will require sustained investment, strong international cooperation, and a balance between technological innovation and societal needs. While challenges persist in data, modeling, and computation, ongoing technological advancements and cross-disciplinary efforts will empower DTO to play a pivotal role in achieving sustainable goals in the UN Ocean Decade.
